# Effects of a 12-week whole-grain or refined wheat intervention on plasma acylcarnitines, bile acids and signaling lipids, and association with liver fat: A *post-hoc* metabolomics study of a randomized controlled trial

**DOI:** 10.3389/fnut.2022.1026213

**Published:** 2022-10-13

**Authors:** Anouk Gijbels, Sophie Schutte, Diederik Esser, Suzan Wopereis, Gerard Bryan Gonzales, Lydia A. Afman

**Affiliations:** ^1^Division of Human Nutrition and Health, Wageningen University and Research, Wageningen, Netherlands; ^2^Research Group Microbiology and Systems Biology, TNO, Netherlands Organization for Applied Scientific Research, Zeist, Netherlands

**Keywords:** whole-grain, wheat, metabolomics, acylcarnitines, bile acids, glycerophospholipids, liver fat, dietary intervention

## Abstract

**Background:**

We previously showed that whole-grain wheat (WGW) consumption had beneficial effects on liver fat accumulation, as compared to refined wheat (RW). The mechanisms underlying these effects remain unclear.

**Objective:**

In this study, we investigated the effects of WGW vs. RW consumption on plasma metabolite levels to explore potential underlying mechanisms of the preventive effect of WGW consumption on liver fat accumulation.

**Methods:**

Targeted metabolomics of plasma obtained from a concluded 12-week double-blind, randomized controlled trial was performed. Fifty overweight or obese men and women aged 45–70 years with mildly elevated levels of plasma cholesterol were randomized to either 98 g/d of WGW or RW products. Before and after the intervention, a total of 89 fasting plasma metabolite concentrations including acylcarnitines, trimethylamine-N-oxide (TMAO), choline, betaine, bile acids, and signaling lipids were quantified by UPLC-MS/MS. Intrahepatic triglycerides (IHTG) were quantified by ^1^H-MRS, and multiple liver markers, including circulating levels of β-hydroxybutyrate, alanine transaminase (ALT), aspartate transaminase (AST), γ-glutamyltransferase (γ-GT), serum amyloid A (SAA), and C-reactive protein, were assessed.

**Results:**

The WGW intervention increased plasma concentrations of four out of 52 signaling lipids—lysophosphatidic acid C18:2, lysophosphatidylethanolamine C18:1 and C18:2, and platelet-activating factor C18:2—and decreased concentrations of the signaling lipid lysophosphatidylglycerol C20:3 as compared to RW intervention, although these results were no longer statistically significant after false discovery rate (FDR) correction. Plasma concentrations of the other metabolites that we quantified were not affected by WGW or RW intervention. Changes in the above-mentioned metabolites were not correlated to change in IHTG upon the intervention.

**Conclusion:**

Plasma acylcarnitines, bile acids, and signaling lipids were not robustly affected by the WGW or RW interventions, which makes them less likely candidates to be directly involved in the mechanisms that underlie the protective effect of WGW consumption or detrimental effect of RW consumption on liver fat accumulation.

**Clinical trial registration:**

[www.ClinicalTrials.gov], identifier [NCT02385149].

## Introduction

In concurrence with the global obesity epidemic, prevalence rates of non-alcoholic fatty liver (NAFL) are on the rise (1). A 2016 study estimated that one in four adults has NAFL worldwide (1). NAFL is defined as excessive hepatic fat accumulation not caused by significant alcohol consumption or other diseases or medication known to induce steatosis (2). NAFL can progress to steatohepatitis (NASH), which is one of the leading causes of chronic liver disease and increases morbidity and mortality from cardiovascular disease (CVD) and type 2 diabetes mellitus (T2DM) (2–6).

Hepatic fat accumulates when hepatic lipid storage exceeds lipid disposal. Sources of lipid influx include dietary fatty acids from intestinally derived chylomicron remnants, circulating free fatty acids (FFA) derived from adipose tissue lipolysis, and newly synthesized lipids from carbohydrates or amino acids by hepatic *de novo* lipogenesis (7). Lipids are cleared from the liver via either mitochondrial β-oxidation or export into the circulation in very low-density lipoprotein (VLDL) particles (7). Key modifiable risk factors for a disequilibrium between hepatic lipid storage and clearance resulting in liver fat accumulation include abdominal obesity, insulin resistance, and dyslipidemia, although the direction of causality between hepatic steatosis and other metabolic abnormalities is unclear and may best be described as bidirectional (2, 6, 8, 9).

The prevention and treatment of hepatic steatosis is primarily based on weight loss (2), although dietary modification has also been shown to affect liver fat independently of weight change (10). A modification that has been suggested to benefit liver health is replacing refined grains with whole grains (11, 12). Compared to refined grains, whole grains contain higher amounts of various nutrients and phytochemicals that may benefit liver health, such as fibers, betaine, and choline (11). Both betaine and choline are directly involved in hepatic lipid metabolism. Choline’s major fate is incorporation into phosphatidylcholine (PC), which is required for packaging and export of lipids from the liver in VLDL, and thereby is essential for hepatic lipid disposal (13). Choline can also be oxidized to betaine. Betaine in turn acts as a methyl-group donor in the methionine-homocysteine cycle in the liver, which plays a central role in *de novo* synthesis of PC (14, 15). Although there are various hypotheses on how whole grain consumption may contribute to liver health, the exact mechanisms are as of yet unknown (11).

We previously performed a randomized, double-blind, parallel trial (16) in 50 overweight individuals and found that 12 weeks of 98 g/d refined wheat (RW) products resulted in a 49% relative increase in intrahepatic triglycerides (IHTG), while IHTG was not affected by whole-grain wheat (WGW) intervention. In the current study, we investigated the effects of the 12-week RW or WGW intervention on plasma levels of metabolites involved in lipid metabolism, i.e. acylcarnitines, trimethylamine-N-oxide (TMAO), choline, betaine, bile acids, and other signaling lipids, in order to explore potential mechanisms that underlie the preventive effect of whole grain consumption on liver fat accumulation.

## Materials and methods

### Study design and participants

**FIGURE 1:**
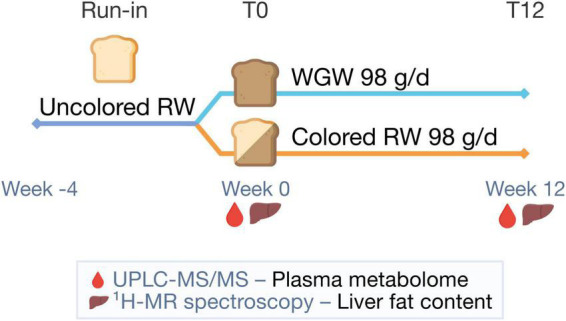
Study design. After a 4-week run-in period with uncolored refined wheat (RW) products, participants were randomized to 12 weeks of 98 g/d whole-grain wheat (WGW) products or RW products. In week 0 (T0) and 12 (T12), the plasma metabolome was measured using targeted ultra-performance liquid chromatography-tandem mass spectrometry (UPLC-MS/MS) and liver fat content was quantified using proton magnetic resonance spectroscopy (^1^H-MRS).

The current study is a *post-hoc* analysis of a 12-week, randomized, double-blind, parallel trial performed from January to July 2015 at Wageningen University and Research, the Netherlands (Figure 1). Details on study procedures have been reported in the original article (16). The study population consisted of men and postmenopausal women aged 45–70 years, with BMI 25–35 kg/m^2^, mildly elevated plasma cholesterol concentrations (> 5 mmol/L), and habitual consumption of bread and cereals. Exclusion criteria were use of cholesterol-lowering medication, gluten intolerance, smoking, alcohol consumption > 21 glasses/week, > 5 kg weight change in the month prior to screening, and history of medical or surgical events that may affect the study outcome. Fifty subjects were included and randomized to the RW or WGW group, with stratification for gender, age, BMI, and cholesterol level.

Before the start of the intervention period, there was a 4-week run-in period with non-colored RW products to reduce variation of WGW intake in the study population at baseline. The primary outcome of the original study was the change in cardiometabolic risk factors in the WGW vs. RW group.

### Intervention

RW or WGW products were provided to participants during the 12-week intervention period to replace their habitual intake: 100 g/d (four slices) of bread and 33.4 g/d of ready-to-eat-cereals, 98 g/d of RW or WGW flour in total. To match the appearance of the WGW products, RW products in the intervention period were colored using roasted wheat malt and caramelized sugar. RW and WGW products had comparable energy content and macronutrient composition, except for fiber content (RW 3.5 g fiber/100 g; WGW 7.8 g fiber/100 g) (16).

All participants were instructed to not consume additional whole-grain products during both the run-in period and the intervention period. Consumption of additional refined grain products was allowed in both groups. Compliance was evaluated by counting the weekly returned intervention product packages and measuring change in total plasma alkylresorcinol concentrations, which is a biomarker for whole-wheat grain intake.

### Intrahepatic triglycerides and other liver parameters

IHTG was quantified by proton magnetic resonance spectroscopy (^1^H-MRS) on a 3T whole-body scanner (Siemens, Munich, Germany) (16). Plasma levels of the ketone body β-hydroxybutyrate were measured by colorimetric assay. Plasma levels of the liver enzymes alanine transaminase (ALT), aspartate transaminase (AST), and γ-glutamyltransferase (γ-GT) were analyzed as described previously (16). Plasma concentrations of the acute-phase proteins serum amyloid A (SAA) and C-reactive protein were measured using immunoassays (16).

### Fasting plasma parameters

Blood samples were drawn after an overnight fast. Plasma glucose, insulin, HbA1c, total cholesterol, HDL cholesterol, triglycerides (TG), and FFA were measured as described previously (16). The Homeostatic Model Assessment for Insulin Resistance (HOMA-IR) was calculated by dividing the product of fasting glucose (mmol/L) and insulin (mU/L) by 22.5.

### Plasma metabolomics

Plasma metabolite levels were measured with two targeted ultra-performance liquid chromatography-tandem mass spectrometry (UPLC-MS/MS) platforms by the Biomedical Metabolomics Facility Leiden (the Netherlands). The acylcarnitine platform covers acylcarnitines as well as betaine, choline, carnitine, and TMAO. The signaling lipid platform covers FFA, lysophospholipids, endocannabinoids, oxylipins, isoprostanes, prostaglandins, and bile acids. Details on the methods used for metabolomic analyses can be found in Supplementary Material. Metabolite levels are expressed as relative response ratios (target area/ISTD area; unit free) to appropriate internal standards. After quality control correction, a total of 26 acylcarnitines, 61 signaling lipids including nine bile acids, and three other metabolites (betaine, choline, TMAO) complied with the acceptance criterium of RSDqc < 15%. Metabolites with ≥ 20% missing values per intervention group were removed from the dataset and metabolite measurements that fell below the limit of detection were imputed with half of the lowest observed level for this metabolite. The bile acid TCA was removed from the dataset due to 20% missing values (RW *n* = 5, WGW *n* = 5). Remaining missing values (*n* = 18 in total) were imputed with half of the lowest observed value for that respective metabolite.

### Statistical analyses

Plasma metabolite levels were log2 transformed and autoscaled before analyses. Intervention effects on plasma metabolite levels were tested using ANCOVA with the post-intervention value as dependent variable and baseline value as covariate. *Q*-values corrected for a false discovery rate (FDR) of 0.05 were calculated using the Benjamin-Hochberg procedure. Within-group changes in individual metabolite levels upon the intervention were tested using paired *t*-tests.

To assess whether changes in metabolite levels upon the intervention were accompanied by changes in liver markers, we calculated Pearson correlation coefficients between changes in metabolite levels and changes in liver markers. We also assessed Pearson correlations between metabolite levels and liver markers at baseline. In addition, partial correlations with adjustment for age, gender, and BMI were calculated. Normality of the liver markers was assessed by visual inspection of residual Q-Q plots and they were log transformed if not normally distributed. All analyses were performed using SPSS version 25 software (IBM Corp.).

## Results

All participants completed the 12-week intervention and plasma metabolite concentrations were measured in samples of all participants (RW *n* = 25, WGW *n* = 25). Baseline characteristics were comparable between the two intervention groups (Table 1). As previously reported, compliance was between 99.5 and 100% based on return of intervention product packages, and 96% of participants could be correctly classified to either the RW or WGW group based on (change in) plasma alkylresorcinol concentrations (16).

**TABLE 1:** Baseline characteristics of the refined wheat (RW) and whole-grain wheat (WGW) groups.

	RW group (*n* = 25)	WGW group (*n* = 25)
Women, *n* (%)	9 (36.0%)	10 (40.0%)
Age, years	61 ± 6	61 ± 5
BMI, kg/m^2^	27.6 ± 2.6	28.0 ± 2.1
HOMA-IR	1.9 (1.2, 2.5)	2.1 (1.2, 3.2)
HbA1c, mmol/mol	37.5 ± 2.3	36.1 ± 3.8
IHTG, %	2.5 (1.6, 6.9)	2.1 (1.7, 7.0)
Total cholesterol, mmol/L	5.8 ± 0.9	5.8 ± 0.7
HDL cholesterol, mmol/L	1.3 ± 0.3	1.3 ± 0.4
Triglycerides, mmol/L	1.5 (1.2, 1.9)	1.5 (1.0, 2.2)
Free fatty acids, mmol/L	0.5 ± 0.2	0.4 ± 0.1

Data are presented as mean ± SD or median (25th percentile, 75th percentile) if not normally distributed.

BMI, body mass index; HOMA-IR, Homeostatic Model Assessment for Insulin Resistance; HbA1c, glycated hemoglobin; IHTG, intrahepatic triglycerides; HDL, high-density lipoprotein.

### Whole-grain wheat vs. refined wheat effects on plasma acylcarnitines, bile acids, and signaling lipids

**FIGURE 2:**
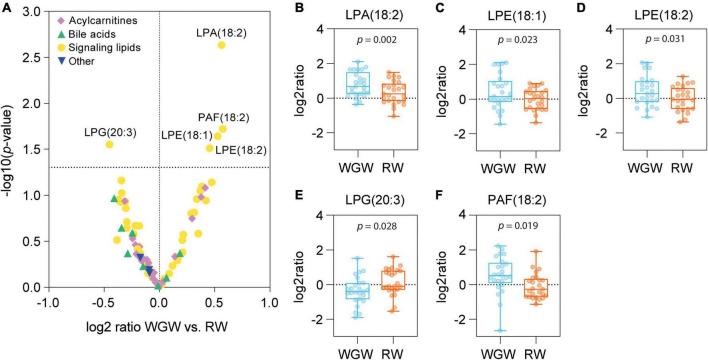
Effects of 12-week whole-grain wheat (WGW) vs. refined wheat (RW) intervention on plasma concentrations of 89 acylcarnitines, bile acids, and signaling lipids. **(A)** Volcano plot in which the differences in changes in individual metabolites between the WGW vs. RW group are plotted against –log 10(*p*-value), as tested by ANCOVA. The horizontal dotted line indicates *p* = 0.05. **(B–F)** Box plots of the change in plasma concentrations of lysophosphatidic acid C18:2 [LPA(18:2)] **(B)**, lysophosphatidylethanolamine C18:1 [LPE(18:1)] **(C)**, lysophosphatidylethanolamine C18:2 [LPE(18:2)] **(D)**, lysophosphatidylglycerol C20:3 [LPG(20:3)] **(E)**, and platelet-activating factor C18:2 [PAF(18:2)] **(F)** upon the WGW (blue) or RW (orange) intervention. The box plots represent the minimum, first quartile, median, third quartile, and maximum. Crude *p*-values are reported for the mean difference between the groups, as tested by ANCOVA.

Plasma concentrations of five out of 52 signaling lipids were changed upon the WGW vs. RW intervention (Figure 2A and Supplementary Table 1). Lysophosphatidic acid C18:2 [LPA(18:2)] was increased upon intervention in both groups, but to a greater extent in the WGW group (log2 ratio mean ± SD: WGW: 0.79 ± 0.70, *p* < 0.001; RW: 0.33 ± 0.66, *p* = 0.018; WGW vs. RW, crude *p* = 0.002) (Figure 2B). Two lysophosphatidylethanolamine (LPE) species, LPE(18:1) and LPE(18:2), as well as platelet-activating factor C18:2 [PAF(18:2)] were increased in the WGW group, while they were not changed in the RW group (Figures 2C,D,F). Lysophosphatidylglycerol C20:3 [LPG(20:3)] was decreased in the WGW group (−0.36 ± 0.81, *p* = 0.036), and unchanged in the RW group (0.15 ± 0.77, *p* = 0.32; WGW vs. RW, crude *p* = 0.028) (Figure 2E). None of these differences remained statistically significant after FDR correction. We found no effects of WGW or RW intervention on plasma concentrations of betaine, choline, or TMAO, nor on the eight plasma bile acids and 26 plasma acylcarnitines that we quantified (Supplementary Table 1).

### Correlations between plasma metabolites and liver markers

**FIGURE 3:**
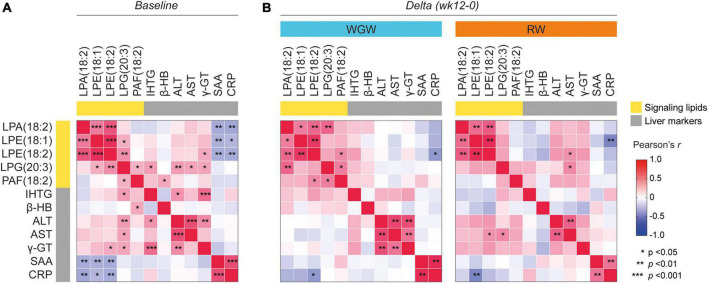
Correlation heatmap of Pearson correlations between plasma levels of LPA(18:2), LPE(18:1), LPE(18:2), LPG(20:3), and PAF(18:2), and liver markers including liver fat (IHTG). **(A)** Correlations at baseline. **(B)** Correlations between changes in LPA(18:2), LPE(18:1), LPE(18:2), LPG(20:3), and PAF(18:2) (expressed as log2 ratios) with changes in liver markers upon the 12-week whole-grain wheat (WGW) (left) or refined wheat (RW) (right) intervention. Asterisks indicate crude *p*-value < 0.05, < 0.01, or < 0.001. LPA(18:2), lysophosphatidic acid C18:2; LPE(18:1), lysophosphatidylethanolamine C18:1; LPE(18:2), lysophosphatidylethanolamine C18:2; LPG(20:3), lysophosphatidylglycerol C20:3; PAF(18:2), platelet-activating factor C18:2; IHTG, intrahepatic triglycerides; β-HB, β-hydroxybutyrate; ALT, alanine transaminase; AST, aspartate transaminase; γ-GT, γ-glutamyltransferase; SAA, serum amyloid A; CRP, C-reactive protein.

As previously reported, 12 weeks of RW intervention resulted in a relative increase of 49% in IHTG, whereas 12 weeks of WGW intervention did not affect IHTG (16). To explore whether plasma levels of the five metabolites that were affected by WGW were related to liver health, we tested correlations between these metabolite levels and liver markers including IHTG, both in response to the 12-week intervention and at baseline. Change in IHTG was not correlated to change in plasma concentrations of any of the five metabolites (Figure 3B). Change in plasma LPE(18:1) was inversely correlated to change in CRP in the RW group (*r* = −0.57, *p* = 0.004), whereas change in plasma LPE(18:2) was inversely correlated to change in CRP in the WGW group (*r* = −0.48, *p* = 0.01). In addition, changes in plasma LPE(18:2) and LPG(20:3) were positively correlated to change in AST in the RW group (*r* = 0.42, *p* = 0.04; *r* = 0.45, *p* = 0.02), but not in the WGW group (*r* = −0.16, *p* = 0.45; *r* = −0.05, *p* = 0.81) (Figure 3B). Performing these correlation analyses in the complete study population while adjusting for intervention group strengthened the inverse correlations between changes in LPA(18:2), LPE(18:1), LPE(18:2) and CRP (*r* = −0.29, *p* = 0.047; *r* = −0.37, *p* = 0.009; *r* = −0.41, *p* = 0.004), and yielded similar results for the other correlations (data not shown).

At baseline, plasma LPA(18:2), LPE(18:1), and LPE(18:2) were inversely correlated to SAA and CRP (*r* = −0.35 to −0.39, *p* < 0.05) (Figure 3A). These correlations, however, were driven by data points from three participants, and exclusion of these data points resulted in a loss of significant correlations (*r* = −0.08 to −0.21, *p* > 0.15) (Supplementary Figure 1). Exclusion of these data points did not affect the correlations between changes in LPA(18:2), LPE(18:1), LPE(18:2) and change in CRP in the analyses in the complete study population or stratified for intervention group (data not shown). Plasma LPG(20:3) was positively correlated to IHTG (*r* = 0.37, *p* = 0.02), as well as to the liver enzymes ALT, AST, and γ-GT (*r* = 0.29–0.38, *p* < 0.05). LPE(18:2) was also positively correlated to γ-GT (*r* = 0.32, *p* = 0.02). PAF(18:2) levels were positively correlated to plasma levels of β-hydroxybutyrate (*r* = 0.33, *p* = 0.019) (Figure 3A). Adjustment for age, gender, and BMI annulled the correlation between change in LPE(18:2) and change in AST, but did not affect the other correlations.

### Correlations between plasma betaine and choline and liver fat

**FIGURE 4:**
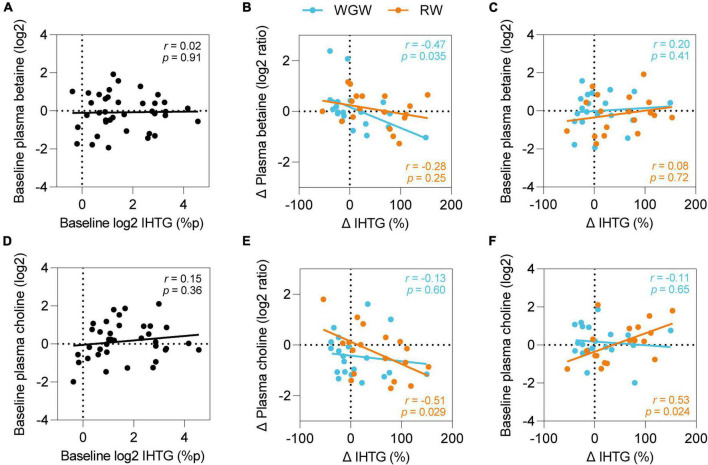
Plasma betaine and choline and effects of RW vs. WGW on IHTG. Pearson correlations between plasma betaine and IHTG at baseline **(A)**, change in plasma betaine and IHTG upon 12-week RW or WGW intervention **(B)**, baseline plasma betaine and change in IHTG **(C)**, plasma choline and IHTG at baseline **(D)**, change in plasma choline and IHTG upon 12-week RW or WGW intervention **(E)**, baseline plasma choline and change in IHTG **(F)**. Adjustment for age, gender, and BMI attenuated the correlation between baseline choline and change in IHTG (*r* = 0.42, *p* = 0.12).

We hypothesized that betaine and choline may be involved in WGW’s protective effect on liver fat accumulation and therefore examined correlations between betaine and choline and IHTG, even though we did not find overall changes in plasma betaine and choline levels upon RW or WGW intervention. At baseline, IHTG was not correlated to plasma betaine or choline (Figures 4A,D). Upon intervention, change in IHTG was inversely correlated to change in plasma choline in the RW group (*r* = −0.51, *p* = 0.03) (Figure 4E) and to change in plasma betaine in the WGW group (*r* = −0.47, *p* = 0.03; Figure 4B), also after adjustment for age, gender, and BMI.

We further explored whether the correlations of change in plasma choline and betaine levels with change in IHTG were dependent on baseline choline or betaine levels. Baseline betaine levels were not correlated to change in IHTG in the WGW group (*r* = 0.08, *p* = 0.72) (Figure 4C), nor did adjustment for baseline levels attenuate the correlation between change in betaine levels and change in IHTG (*r* = −0.47, *p* = 0.04). In the RW group, baseline choline levels were positively correlated to change in IHTG upon the intervention (*r* = 0.53, *p* = 0.02) (Figure 4F), and correction for baseline levels annulled the correlation with change in choline levels (*r* = −0.33, *p* = 0.19). Adjustment for age, gender, and BMI attenuated the correlation between baseline choline and change in IHTG (*r* = 0.42, *p* = 0.12).

## Discussion

We previously showed that WGW consumption prevented liver fat accumulation, as compared to RW in overweight or obese individuals. Here, we investigated the effects of this 12-week WGW vs. RW intervention on plasma concentrations of a total of 89 acylcarnitines, bile acids, and signaling lipids with the aim to explore potential underlying mechanisms of the preventive effect of WGW consumption on liver fat accumulation. The WGW intervention affected plasma concentrations of five metabolites that belong to the (lyso)glycerophospholipid (GPL) class (17): WGW increased plasma platelet-activating factor C18:2 [PAF(18:2)], lysophosphatidylethanolamine C18:1 [LPE(18:1)], lysophosphatidylethanolamine C18:2 [LPE(18:2)], and lysophosphatidic acid C18:2 [LPA(18:2)], and decreased plasma lysophosphatidylglycerol C20:3 [LPG(20:3)], as compared to RW intervention. These results, however, were no longer statistically significant after FDR correction. The change in liver fat upon 12 weeks of RW or WGW was not accompanied by changes in levels of either of these five metabolites.

Whole grains have previously been reported to affect plasma levels of several GPLs. Compared to refined grains, whole grain intervention has been found to reduce plasma levels of the choline derivative glycerophosphocholine (GPC) (18), and increase plasma levels of various lysophosphatidylcholine (LPC) and phosphatidylcholine (PC) species (19, 20). To our knowledge, no prior studies have reported the effects of whole grain intervention on the specific plasma GPL that we measured.

WGW intervention increased circulating levels of PAF(18:2), while 12 weeks of RW did not affect PAF. PAF is a glycerophosphocholine that mediates a broad range of biological actions. It is produced by platelets, endothelial cells, and immune cells in response to various stimuli and is primarily known for its pro-inflammatory actions (21–23). Circulating PAF has been reported to be elevated in CVD and various inflammatory diseases in humans (24–26). Accordingly, the observed increase in plasma PAF upon WGW intervention may point toward increased inflammation, but this is unlikely since the WGW intervention actually tended to decrease the markers of systemic inflammation CRP and SAA, as we previously reported (16). Paradoxically, deficiency of the PAF receptor in mice fed a high-fat or high-carbohydrate diet has been found to impair metabolic health and increase liver fat accumulation, which indicates that PAF signaling may be required for maintenance of metabolic health in diet-induced obesity (27–32). The ramifications of the increase in plasma PAF levels upon WGW thus remain elusive.

In addition, WGW increased plasma LPE(18:1) and LPE(18:2), compared to RW. The physiological functions of LPEs are largely unknown (33). Individuals with NAFLD have been reported to have lower circulating LPE(18:1) and LPE(18:2) levels compared to healthy controls (34), and plasma LPE(18:1) and LPE(18:2) have been inversely associated with incident T2DM (35). We observed inverse correlations between change in these plasma LPEs and change in CRP upon the interventions, indicating that increases in these LPEs were accompanied by a reduction in systemic inflammation. Although no significant correlations with changes in liver fat were observed, the WGW-induced increase in plasma LPE(18:1) and LPE(18:2) may point toward a potential lead for the protective role of WGW on liver fat accumulation.

Both the WGW and RW interventions increased circulating LPA(18:2) levels, but WGW resulted in a larger increase. Circulating LPA is primarily generated by the adipose tissue-derived enzyme autotaxin, which hydrolyzes LPC and other lysophospholipids into LPA in the circulation (36). Serum autotaxin levels have been reported to be elevated in hepatic steatosis and (hepatic) inflammatory diseases (37–40). Although actual LPA levels were not measured in the majority of these cross-sectional studies, it can be expected that LPA levels are similarly elevated in these conditions, since serum autotaxin levels are strongly positively correlated to plasma LPA levels (37, 41). In mice, heterozygous knockout of ATX, resulting in 50% reduced circulating LPA levels, has been reported to mitigate high-fat diet-induced liver fat accumulation and inflammation (42). In contrast to these findings, we observed an increase in plasma LPA(18:2) and prevention of liver fat accumulation upon WGW consumption, as compared to RW, as well as an inverse correlation between change in plasma LPA(18:2) and change in CRP, with increases in LPA(18:2) thus being accompanied by a reduction in systemic inflammation. Hence, LPA seems to be implicated in inflammation and hepatic lipid metabolism, but the cause and interpretation of the increases in plasma levels we observed after WGW intervention remain as of yet unclear.

Compared to the RW intervention, the WGW intervention decreased plasma concentrations of LPG(20:3). LPG is a precursor of *de novo* synthesis of phosphatidylglycerol, a phospholipid that is mainly abundant in lung surfactant (33, 43). Very little is known about its biological actions (33, 43). At baseline, plasma LPG(20:3) was positively correlated to IHTG and the liver enzymes ALT, AST, and γ-GT, but the decrease in LPG(20:3) upon the WGW intervention was not accompanied by change in liver fat nor liver enzymes. It thus appears that plasma LPG might be related to liver health and/or function, although the nature of the association is unclear since we did not observe parallel changes in LPG and liver fat upon WGW. This lack of correlation may also be partly due to limited power after stratification for intervention group. Future studies with large sample size are required to clarify the potential role of LPG in liver fat accumulation.

We hypothesized that choline and betaine may be involved in the protective effect of WGW on liver fat accumulation given their roles in TG secretion from the liver into the circulation as VLDL and hepatic one-carbon metabolism, respectively. Plasma choline was not significantly different between WGW vs. RW intervention, which is in line with findings from a cross-over trial that tested 8-week interventions with either 50 g/1,000 kcal whole grains or refined grains in 33 overweight or obese individuals (44). We also did not observe changes in plasma betaine upon WGW or RW. Various other studies did report increased plasma betaine levels after whole grain intervention (45–47), although not all (18, 44, 48). This incongruency seems to arise primarily from differences in whole grain dose: the studies that reported increases in betaine levels used 200–485 g/d of whole-grain cereals or bread (45–47), which is considerably more than the 133 g/d in our study.

Interestingly, we did observe that an increase in plasma betaine upon the WGW intervention was correlated to a reduction in liver fat. The steatosis-lowering potential of betaine, however, remains controversial since the beneficial effects of betaine supplementation that have been observed in animal studies (14) have only been reproduced in humans in a small pilot study in 10 NASH patients, (49) and not in recent clinical trials in individuals with NAFLD or prediabetes (50, 51). It could be speculated that the effects of increasing betaine intake with betaine-rich foods or supplements on liver fat may depend on individual characteristics such as sex, BMI, health status, habitual diet, and other factors, since such factors appear to mediate the effects of betaine on cardiovascular risk factors (52) and the same may be the case for effects of betaine on liver fat.

Strengths of this study include the relatively long intervention period of 12 weeks, which enabled us to study longer-term rather than acute effects of WGW and RW consumption. In addition, we included a 4-week run-in period with RW for all participants to reduce variation in the study population at the start. Both researchers and participants were blinded to the intervention by coloring RW products to match the appearance of WGW products. Compliance to the intervention based on recall of empty product packages and plasma alkylresorcinol levels was high and all participants completed the study.

This study may be limited by its relatively small sample size, which was originally determined to detect changes in plasma cholesterol levels. Given the large interindividual variation in plasma metabolite concentrations—both at baseline and in response to a 12-week diet—we may have missed effects of the WGW or RW intervention on plasma metabolite levels due to insufficient power. In addition, the five metabolites that we identified to be differentially affected by WGW compared to RW intervention in this explorative study were no longer statistically significantly different after FDR correction. There is a possibility that these findings are chance findings and they need to be confirmed in larger studies.

## Conclusion

In conclusion, in this *post-hoc* analysis of a double-blind, randomized controlled trial investigating the effects of 12 weeks of WGW or RW intervention on plasma acylcarnitines, bile acids, and signaling lipids in middle-aged, overweight adults, we observed that plasma concentrations of five signaling lipids involved in glycerophospholipid metabolism were altered upon WGW as compared to RW intervention, but these changes did not remain statistically significant after FDR correction. The changes in plasma concentrations of these five signaling lipids upon the intervention were not correlated to changes in liver fat, which makes these metabolites less likely candidates to be involved in the mechanisms underlying the protective effect of WGW consumption or detrimental effect of RW consumption on liver fat accumulation.

## Data availability statement

The datasets presented in this article are not readily available because participants did not provide consent for their data to be shared publicly. Requests to access the datasets should be directed to the corresponding author.

## Ethics statement

The studies involving human participants were reviewed and approved by the Medical Ethics Committee of Wageningen University. The patients/participants provided their written informed consent to participate in this study.

## Author contributions

DE, SW, and LA designed the study. SS and DE coordinated and performed the execution of the trial. LA supervised execution of the trial. AG analyzed the data and wrote the draft manuscript. GG and LA critically revised the draft. All authors read and approved the final manuscript.
