# Supplementary material for: Effects of a 12-week whole-grain or refined wheat intervention on plasma acylcarnitines, bile acids and signaling lipids, and association with liver fat: A post-hoc metabolomics study of a randomized controlled trial
Source: Front Nutr. 2022 Oct 13;9:1026213. doi: 10.3389/fnut.2022.1026213 (PMC9624226; doi:10.3389/fnut.2022.1026213)
Supplement: Supplementary file 1 [file Data_Sheet_1.pdf]

1 Metabolomics Methods

Plasma metabolite levels were measured with two targeted (UP)LC-MS/MS platforms by the Biomedical Metabolomics Facility Leiden (the Netherlands). All samples were randomized and run in two batches which included calibration lines, quality control (QC) samples and blanks. QC samples were analyzed every 10 samples and were used to assess data quality and to correct for instrument response. Blanks were used to check for background signal compared to the study samples.

The acylcarnitine platform covers acylcarnitines as well as betaine, choline, carnitine, and trimethyl-amine-n-oxide. Ten µL of each sample was spiked with an internal standard solution. Proteins were precipitated by the addition of methanol, after which the supernatant was transferred to an autosampler vial. The vials were transferred to an autosampler tray and cooled to 10°C until the injection. One µL of the sample mixture was injected into the UPLCMS/MS. Chromatographic separation was achieved by UPLC (Agilent 1290, San Jose, CA, USA) on an Accq-Tag Ultra column (Waters). The UPLC was coupled to electrospray ionization on a triple quadrupole mass spectrometer (Agilent 6460, San Jose, CA, USA). Analytes were detected in the positive ion mode and monitored in Multiple Reaction Monitoring (MRM) using nominal mass resolution. Acquired data were evaluated using Agilent MassHunter Quantitative Analysis software (Agilent, Version B.05.01), by integration of assigned MRM peaks and normalization using proper internal standards. The closest-eluting internal standard was employed. In-house developed algorithms were applied using the pooled QC samples to compensate for shifts in the sensitivity of the mass spectrometer over the batches.

The signaling lipid platform covers free fatty acids, lysophospholipids, endocannabinoids, oxylipins, isoprostanes, prostaglandins, and bile acids. The signaling lipids platform is divided in two chromatographic methods: low and high pH. In the low pH method, isoprostanes, prostaglandins, nitro-fatty acids, lysosphingolipids, endocannabinoids, and bile acids are analyzed. The high pH method covers lysosphingolipids, lysophosphatidic acids, lysophosphatidylglycerol, lyso-phosphatidylinositol, lysophosphatidyserine, lysophosphatidylethanolamines, cyclic-phosphatidic acids and fatty acids. Each sample was spiked with antioxidant and internal standard solution. The extraction of the compounds was performed via liquid-liquid extraction. To extract the analytes from the aqueous phase, butanol and methyl tert-butyl ether were used. After collection, the organic phase was concentrated by first drying and then reconstituted in a smaller volume. After reconstitution, the extract was transferred into amber autosampler vials and used for high and low pH injection. A Shimadzu system formed by three high pressure pumps (LC-30AD), controller (CBM-20Alite), auto sampler (SIL-30AC) and an oven (CTO-30A) from Shimadzu Benelux, was coupled online with a LCMS-8050 triple quadrupole mass spectrometer (Shimadzu) for high pH measurements. A LCMS-8060 triple quadrupole mass spectrometer (Shimadzu) was coupled to the Shimadzu system for low pH measurements. Both systems were operated using LabSolutions data acquisition software (Version 5.89, Shimadzu). An Acquity UPLC BEH C18 column (Waters) was used to measure the samples in the low pH method. For the high pH method, a Kinetex EVO column by Phenomenex was used. The triple quadrupole mass spectrometer was used in polarity switching mode and all analytes were monitored in dynamic MRM. The acquired data was evaluated using LabSolutions Insight software (Version 3.3, Shimadzu), by integration of assigned MRM peaks and normalization using accordingly selected internal standards. When available, a deuterated version of the target compound was used as internal standard. For the other compounds, the closest-eluting internal standard was employed. Blank samples were used to check blank levels.

2 Supplementary Table

Supplementary Table 1. Effects of 12 weeks of refined wheat (RW) or whole-grain wheat (WGW) intervention on plasma acylcarnitines, bile acids, and signaling lipids.

	Refined wheat group (n = 25)					Whole-grain wheat group (n = 25)					RW vs. WGW	
	**Baseline[a]**		**log2 ratio wk12-0**		***p*-value[b]**	**Baseline[a]**		**log2 ratio wk12-0**		***p*-value[b]**	***p*-value[c]**	**FDR *q*-value[d]**
**Acylcarnitines**												
2-methylbutyroylcarnitine	0.125	± 0.925	0.091	± 0.633	0.479	-0.253	± 1.133	0.165	± 0.698	0.249	0.861	0.946
Acetylcarnitine	0.011	± 1.127	0.143	± 1.105	0.524	-0.221	± 1.085	0.276	± 1.213	0.267	0.886	0.950
Butyrylcarnitine	0.049	± 1.071	0.117	± 0.385	0.142	-0.143	± 0.864	0.072	± 0.488	0.467	0.691	0.867
Carnitine	-0.148	± 1.024	0.385	± 0.701	0.011	-0.064	± 1.079	0.040	± 0.865	0.821	0.116	0.605
Decanoylcarnitine	0.494	± 0.890	-0.635	± 0.666	<0.001	0.111	± 1.048	-0.575	± 0.800	0.001	0.730	0.855
Decenoylcarnitine	0.479	± 0.908	-0.487	± 0.756	0.004	-0.012	± 0.930	-0.448	± 0.893	0.019	0.607	0.806
Dodecenoylcarnitine	0.554	± 0.939	-0.628	± 0.810	0.001	0.072	± 0.940	-0.626	± 0.945	0.003	0.345	0.830
Hexadecenoylcarnitine	0.162	± 0.882	-0.178	± 0.770	0.259	0.086	± 1.031	-0.317	± 0.999	0.125	0.491	0.809
Hexanoylcarnitine	0.251	± 0.915	-0.174	± 0.561	0.133	-0.111	± 0.935	-0.106	± 0.767	0.497	0.866	0.940
Isobutyrylcarnitine	0.037	± 1.124	0.006	± 0.476	0.951	-0.054	± 1.120	0.028	± 0.644	0.831	0.962	0.984
Isovalerylcarnitine	-0.382	± 0.936	0.896	± 0.737	<0.001	-0.396	± 0.862	0.659	± 1.010	0.003	0.295	0.773
Lauroylcarnitine	0.495	± 0.849	-0.578	± 0.783	0.001	0.065	± 1.095	-0.541	± 0.921	0.007	0.488	0.819
Linoleylcarnitine	-0.126	± 0.889	-0.135	± 0.738	0.368	0.174	± 1.136	0.041	± 0.944	0.830	0.179	0.691
Malonylcarnitine	0.047	± 1.062	0.065	± 1.060	0.761	-0.027	± 0.901	-0.105	± 1.068	0.629	0.435	0.841
Myristoilcarnitine	0.155	± 0.895	-0.245	± 0.795	0.137	0.171	± 1.038	-0.407	± 0.816	0.020	0.471	0.856
Nonaylcarnitine	0.016	± 1.016	0.032	± 0.419	0.703	-0.002	± 1.077	-0.060	± 0.739	0.687	0.543	0.779
Octanoylcarnitine	0.452	± 0.887	-0.536	± 0.631	<0.001	0.031	± 1.024	-0.432	± 0.783	0.011	0.979	0.979
Octenoylcarnitine	0.023	± 1.013	0.070	± 0.604	0.565	-0.200	± 1.077	0.283	± 0.859	0.112	0.462	0.875
Oleylcarnitine	-0.119	± 0.894	-0.198	± 0.939	0.303	0.235	± 1.229	-0.034	± 1.095	0.877	0.104	0.774
Palmitoylcarnitine	-0.143	± 0.902	0.081	± 0.751	0.597	0.145	± 1.119	-0.083	± 0.960	0.670	0.804	0.906
Propionylcarnitine	-0.210	± 1.289	0.403	± 0.911	0.037	-0.072	± 0.825	0.162	± 0.752	0.292	0.363	0.829
Stearoylcarnitine	-0.075	± 0.874	-0.165	± 0.939	0.389	0.066	± 1.142	0.183	± 0.937	0.339	0.083	0.818
Tetradecadienylcarnitine	0.461	± 0.841	-0.580	± 0.846	0.002	0.127	± 1.038	-0.594	± 1.022	0.008	0.479	0.835
Tetradecenoylcarnitine	0.363	± 0.793	-0.470	± 0.798	0.007	0.137	± 1.077	-0.531	± 1.082	0.022	0.514	0.776
Tiglylcarnitine	0.218	± 0.799	-0.034	± 0.708	0.812	-0.239	± 1.110	0.076	± 0.921	0.682	0.899	0.953
Valerylcarnitine	0.096	± 1.158	0.139	± 0.564	0.230	-0.221	± 0.913	0.112	± 0.718	0.445	0.501	0.811

Supplementary Table 1. (continued)

	Refined wheat group (n = 25)			Whole-grain wheat group (n = 25)			RW vs. WGW	
	Baseline[a]	log2 ratio wk12-0	*p*-value[b]	Baseline[a]	log2 ratio wk12-0	*p*-value[b]	*p*-value[c]	FDR *q*-value[d]
**Bile acids**								
Cholic acid	-0.191 ± 0.983	0.023 ± 0.956	0.905	0.135 ± 0.988	0.088 ± 0.787	0.581	0.427	0.863
Glycochenodeoxycholic acid	-0.136 ± 0.620	0.203 ± 0.611	0.110	0.107 ± 0.869	-0.146 ± 1.221	0.555	0.226	0.746
Glycodeoxycholic acid	-0.233 ± 0.868	0.398 ± 0.781	0.018	0.078 ± 0.938	-0.088 ± 0.983	0.657	0.108	0.736
Glycolithocholic acid	-0.151 ± 0.995	0.340 ± 0.990	0.098	-0.090 ± 1.072	0.142 ± 1.515	0.642	0.591	0.810
Glycoursodeoxycholic acid	-0.304 ± 0.701	0.601 ± 1.751	0.099	-0.070 ± 0.578	0.147 ± 0.627	0.252	0.428	0.847
Lithocholic acid sulphate	-0.180 ± 1.211	0.195 ± 0.978	0.329	0.169 ± 0.790	-0.172 ± 0.617	0.176	0.256	0.786
Taurodeoxycholic acid	-0.171 ± 0.994	0.158 ± 0.717	0.280	0.023 ± 1.033	0.139 ± 1.088	0.528	0.788	0.899
Taurohyodeoxycholic acid	-0.156 ± 0.953	0.190 ± 0.876	0.289	0.007 ± 1.020	0.108 ± 1.037	0.606	0.962	0.995
**Signaling lipids**								
12,13-dihydroxy-9Z-octadecenoic acid	-0.028 ± 0.811	-0.238 ± 0.492	0.024	0.327 ± 1.072	-0.360 ± 0.720	0.020	0.654	0.844
Adrenic acid	0.023 ± 1.170	0.131 ± 0.707	0.364	0.034 ± 0.706	-0.245 ± 0.986	0.226	0.112	0.662
Anandamide	0.041 ± 1.123	-0.099 ± 0.779	0.532	0.279 ± 1.004	-0.540 ± 0.878	0.005	0.095	0.767
Cyclic-Lysophosphatidic acid (18:0)	-0.040 ± 0.646	-0.294 ± 1.375	0.295	0.162 ± 0.658	0.051 ± 0.620	0.683	0.261	0.775
Cortisol	0.164 ± 0.943	-0.169 ± 0.740	0.264	-0.167 ± 0.984	0.174 ± 1.145	0.456	0.419	0.909
Docosahexaenoic acid	0.196 ± 0.927	-0.212 ± 0.546	0.064	0.100 ± 1.090	-0.379 ± 0.517	0.001	0.215	0.736
Docosapentaenoic acid	0.131 ± 0.997	-0.248 ± 0.587	0.045	0.189 ± 0.866	-0.392 ± 0.873	0.034	0.521	0.760
Linoleoyl ethanolamide	0.004 ± 1.000	-0.318 ± 0.891	0.087	0.097 ± 0.877	0.115 ± 1.072	0.596	0.072	0.920
Linoleic acid	0.220 ± 0.931	-0.198 ± 0.584	0.103	0.038 ± 1.036	-0.317 ± 0.998	0.125	0.421	0.892
Lysophosphatidic acid (14:0)	-0.332 ± 1.152	0.670 ± 1.068	0.004	-0.122 ± 1.082	0.239 ± 0.774	0.135	0.117	0.577
Lysophosphatidic acid (16:0)	-0.497 ± 0.693	0.527 ± 0.693	0.001	0.055 ± 1.089	0.357 ± 1.016	0.092	0.932	0.976
Lysophosphatidic acid (16:1)	-0.159 ± 0.972	0.237 ± 0.668	0.088	-0.010 ± 1.055	0.100 ± 0.712	0.488	0.588	0.818
Lysophosphatidic acid (18:1)	-0.569 ± 0.648	0.293 ± 0.699	0.047	0.186 ± 1.030	0.474 ± 0.756	0.004	0.080	0.890
Lysophosphatidic acid (18:2)	-0.448 ± 0.781	0.331 ± 0.655	0.018	-0.111 ± 1.106	0.787 ± 0.691	0.000	0.002	0.207
Lysophosphatidic acid (22:6)	-0.153 ± 0.983	0.085 ± 0.685	0.539	-0.017 ± 1.030	0.253 ± 0.823	0.137	0.307	0.759
Lysophosphatidylethanolamine (16:0)	-0.138 ± 0.894	-0.047 ± 0.561	0.681	0.051 ± 1.012	0.221 ± 0.971	0.266	0.153	0.650
Lysophosphatidylethanolamine (16:1)	-0.088 ± 1.045	0.099 ± 0.699	0.484	0.054 ± 0.984	-0.030 ± 0.850	0.861	0.672	0.854
Lysophosphatidylethanolamine (18:0)	-0.114 ± 0.844	-0.039 ± 0.567	0.735	0.006 ± 1.171	0.256 ± 0.984	0.205	0.110	0.697
Lysophosphatidylethanolamine (18:1)	-0.241 ± 0.885	-0.022 ± 0.614	0.861	0.044 ± 0.975	0.417 ± 0.988	0.045	0.023	0.680
Lysophosphatidylethanolamine (18:2)	-0.144 ± 0.970	-0.013 ± 0.705	0.927	-0.054 ± 1.055	0.410 ± 0.876	0.028	0.031	0.550
Lysophosphatidylethanolamine (18:3)	-0.239 ± 1.007	0.185 ± 1.160	0.434	0.049 ± 1.097	0.195 ± 0.783	0.226	0.481	0.822
Lysophosphatidylethanolamine (20:3)	-0.092 ± 0.904	0.012 ± 0.843	0.945	0.022 ± 1.069	0.129 ± 0.960	0.508	0.501	0.797
Lysophosphatidylethanolamine (20:4)	0.015 ± 0.902	-0.202 ± 0.626	0.119	0.000 ± 1.082	0.172 ± 0.951	0.374	0.089	0.794
Lysophosphatidylethanolamine (20:5)	-0.139 ± 1.006	0.160 ± 0.744	0.293	-0.058 ± 1.184	0.235 ± 1.107	0.299	0.584	0.825
Lysophosphatidylethanolamine (22:4)	-0.060 ± 0.938	0.013 ± 0.665	0.920	-0.024 ± 1.079	0.155 ± 0.902	0.400	0.471	0.873

Supplementary Table 1. (continued)

	Refined wheat group (n = 25)			Whole-grain wheat group (n = 25)			RW vs. WGW	
	Baseline[a]	log2 ratio wk12-0	*p*-value[b]	Baseline[a]	log2 ratio wk12-0	*p*-value[b]	*p*-value[c]	FDR *q*-value[d]
Lysophosphatidylethanolamine (22:5)	-0.081 ± 0.958	-0.017 ± 0.766	0.913	0.041 ± 1.010	0.097 ± 1.096	0.662	0.508	0.780
Lysophosphatidylethanolamine (22:6)	0.051 ± 0.913	-0.184 ± 0.530	0.095	0.021 ± 1.137	0.040 ± 0.896	0.824	0.267	0.742
Lysophosphatidylglycerol (16:0)	-0.063 ± 0.913	0.050 ± 0.750	0.740	0.109 ± 1.059	-0.142 ± 0.846	0.410	0.506	0.790
Lysophosphatidylglycerol (16:1)	0.056 ± 1.189	0.016 ± 0.864	0.927	0.109 ± 0.860	-0.345 ± 0.538	0.004	0.069	1.024
Lysophosphatidylglycerol (18:0)	-0.067 ± 0.907	0.103 ± 0.562	0.368	-0.027 ± 1.043	0.084 ± 0.925	0.653	0.964	0.975
Lysophosphatidylglycerol (18:1)	0.038 ± 0.871	-0.123 ± 0.711	0.396	0.291 ± 1.005	-0.535 ± 0.875	0.005	0.118	0.553
Lysophosphatidylglycerol (18:2)	-0.080 ± 1.031	-0.042 ± 0.753	0.782	0.240 ± 0.849	-0.278 ± 0.774	0.085	0.423	0.875
Lysophosphatidylglycerol (20:3)	-0.036 ± 1.066	0.154 ± 0.768	0.324	0.139 ± 1.019	-0.360 ± 0.811	0.036	0.028	0.625
Lysophosphatidylglycerol (20:4)	0.041 ± 1.108	-0.152 ± 0.729	0.308	0.301 ± 0.819	-0.533 ± 0.943	0.009	0.195	0.723
Lysophosphatidylinositol (16:0)	0.009 ± 1.030	0.234 ± 0.770	0.141	-0.153 ± 1.002	0.054 ± 0.817	0.745	0.263	0.755
Lysophosphatidylinositol (16:1)	0.022 ± 1.113	0.190 ± 0.749	0.217	-0.073 ± 0.887	-0.087 ± 0.769	0.579	0.138	0.614
Lysophosphatidylinositol (18:0)	0.176 ± 0.880	0.075 ± 0.701	0.598	-0.215 ± 1.100	0.004 ± 0.806	0.981	0.360	0.844
Lysophosphatidylinositol (18:1)	-0.136 ± 1.058	0.221 ± 0.686	0.121	-0.099 ± 1.095	0.249 ± 0.747	0.109	0.815	0.907
Lysophosphatidylinositol (18:2)	-0.165 ± 0.869	0.310 ± 0.846	0.080	-0.020 ± 1.117	0.061 ± 0.871	0.727	0.384	0.853
Lysophosphatidylinositol (20:4)	0.070 ± 1.019	0.038 ± 0.898	0.835	-0.103 ± 0.904	0.026 ± 0.850	0.878	0.784	0.906
Lysophosphatidylinositol (22:4)	0.132 ± 1.086	-0.262 ± 0.963	0.186	0.034 ± 0.836	-0.069 ± 0.859	0.692	0.515	0.764
Lysophosphatidylserine (18:0)	-0.067 ± 0.904	-0.135 ± 0.605	0.277	0.084 ± 1.149	0.100 ± 0.917	0.591	0.157	0.637
Lysophosphatidylserine (20:4)	0.034 ± 1.218	-0.176 ± 1.367	0.525	-0.126 ± 0.858	0.361 ± 0.987	0.080	0.114	0.634
Oleic acid	0.187 ± 0.955	-0.166 ± 0.506	0.115	-0.083 ± 0.816	-0.042 ± 1.040	0.842	0.712	0.845
Osbond acid	0.213 ± 1.126	-0.263 ± 0.748	0.091	0.194 ± 0.704	-0.551 ± 1.038	0.014	0.227	0.722
Platelet activating factor (18:2)	-0.026 ± 1.019	-0.102 ± 0.729	0.491	-0.193 ± 0.940	0.540 ± 1.069	0.019	0.019	0.849
Prostaglandin F2α	0.044 ± 0.977	-0.106 ± 0.949	0.582	0.128 ± 0.973	-0.237 ± 0.553	0.042	0.603	0.813
Sphingosine-1-phosphate (16:1)	0.154 ± 0.348	0.053 ± 0.167	0.126	0.007 ± 0.366	-0.376 ± 1.794	0.305	0.306	0.778
Sphingosine-1-phosphate (18:0)	-0.143 ± 1.014	0.096 ± 0.929	0.609	0.169 ± 0.886	-0.148 ± 1.108	0.511	0.704	0.847
Sphingosine-1-phosphate (18:1)	-0.208 ± 1.184	0.193 ± 0.953	0.322	0.126 ± 0.817	-0.030 ± 0.805	0.856	0.695	0.859
Sphingosine-1-phosphate (18:2)	-0.169 ± 1.069	0.149 ± 0.663	0.273	0.162 ± 0.935	-0.133 ± 0.595	0.275	0.214	0.762
Virodhamine	0.039 ± 0.911	-0.044 ± 0.844	0.795	0.141 ± 1.108	-0.317 ± 0.797	0.058	0.271	0.730
**Other**								
Betaine	-0.117 ± 1.058	0.233 ± 0.717	0.117	-0.064 ± 0.910	0.128 ± 0.767	0.414	0.646	0.846
Choline	0.239 ± 1.057	-0.294 ± 1.022	0.163	0.117 ± 0.871	-0.418 ± 0.842	0.020	0.476	0.848
Trimethylamine N-oxide	-0.020 ± 0.898	0.167 ± 0.985	0.404	-0.130 ± 1.194	0.134 ± 0.977	0.501	0.700	0.854

[a] Values are log2-transformed and autoscaled relative response ratios
[b] Within-group changes in individual metabolite levels upon the intervention were tested using paired t-tests
[c] Between-group differences in effects on plasma metabolite levels were tested using ANCOVA with the post-intervention value as dependent variable and baseline value as covariate
[d] Q-values corrected for a false discovery rate (FDR) of 0.05 were calculated using the Benjamin-Hochberg procedure

3 Supplementary Figure

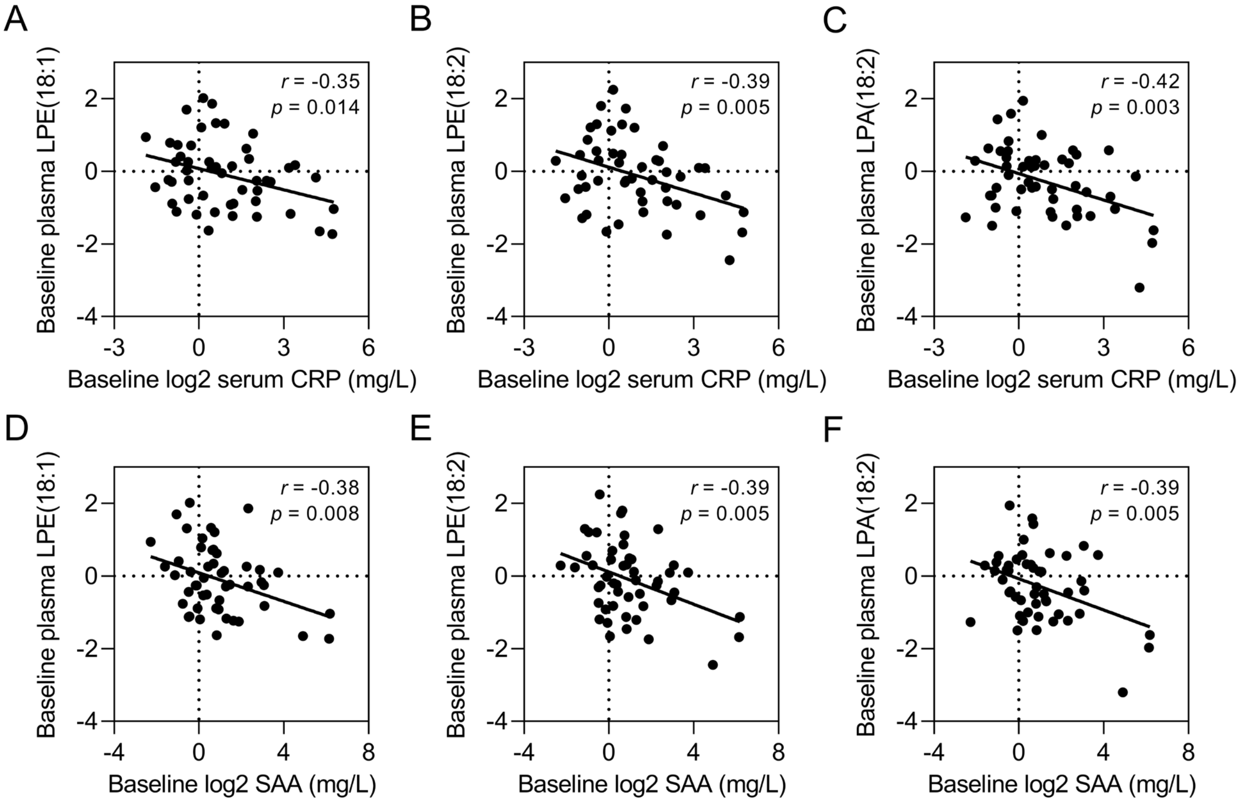

Supplementary Figure 1. Scatter plots with Pearson correlations between baseline plasma LPE(18:1), LPE(18:2), LPA(18:2) and serum C-reactive protein (CRP) (**A-C**), and LPE(18:1), LPE(18:2), LPA(18:2) and serum amyloid A concentrations (SAA) (**D-F**). These correlations were driven by data points from three participants, and exclusion of these data points resulted in a loss of significant correlations (r = -0.08 to -0.21, p > 0.15).
